# Effects of Blue-Light-Induced Free Radical Formation from Catechin Hydrate on the Inactivation of *Acinetobacter baumannii,* Including a Carbapenem-Resistant Strain

**DOI:** 10.3390/molecules23071631

**Published:** 2018-07-04

**Authors:** Meei-Ju Yang, Yi-An Hung, Tak-Wah Wong, Nan-Yao Lee, Jeu-Ming P. Yuann, Shiuh-Tsuen Huang, Chun-Yi Wu, Iou-Zen Chen, Ji-Yuan Liang

**Affiliations:** 1 Tea Research and Extension Station, Taoyuan 32654, Taiwan; 762204@gmail.com; 2 Department of Biotechnology, Ming-Chuan University, Gui-Shan 33343, Taiwan; amyhung840809@gmail.com (Y.-A.H.); jyuann@mail.mcu.edu.tw (J.-M.P.Y.); kevin1060755@gmail.com (C.-Y.W.); 3 Department of Dermatology, Department of Biochemistry and Molecular Biology, National Cheng Kung University Hospital, College of Medicine, National Cheng Kung University, Tainan 70101, Taiwan; dr.kentwwong@gmail.com; 4 Division of Infection, Department of Internal Medicine, National Cheng Kung University Hospital, College of Medicine, National Cheng Kung University, Tainan 70101, Taiwan; nanyao@mail.ncku.edu.tw; 5 Department of Science Education and Application, National Taichung University of Education, Taichung 40306, Taiwan; hstsuen@mail.ntcu.edu.tw; 6 Department of Horticulture and Landscape Architecture, National Taiwan University, Taipei 10617, Taiwan

**Keywords:** ascorbic acid, blue light, catechin, CRAB, inactivation

## Abstract

Catechin is a flavan-3-ol, a derivative of flavans, with four phenolic hydroxyl groups, which exhibits a wide range of physiological properties. Chromatographic analyses were employed to examine the effects of blue light irradiation on the changes of catechin hydrate in an alkaline condition. In particular, the detection of a superoxide anion radical (O_2_•^−^), a reactive oxygen species (ROS), and the inactivation of *Acinetobacter baumannii* (*A. baumannii*)—including a carbapenem-resistant *A. baumannii* (CRAB)—was investigated during the photoreaction of catechin hydrate. Following basification with blue light irradiation, the transparent solution of catechin hydrate turned yellowish, and a chromogenic catechin dimer was separated and identified as a proanthocyanidin. Adding ascorbic acid during the photolytic treatment of catechin hydrate decreased the dimer formation, suggesting that ascorbic acid can suppress the photosensitive oxidation of catechin. When catechin hydrate was irradiated by blue light in an alkaline solution, O_2_•^−^ was produced via photosensitized oxidation, enhancing the inactivation of *A. baumannii* and CRAB. The present findings on the photon-induced oxidation of catechin hydrate provides a safe practice for the inactivation of environmental microorganisms.

## 1. Introduction

Phenolic compounds are the products of the secondary metabolism in plants and are important antioxidants in plants and plant-based foods [1]. Polyphenols contain aromatic rings, with each ring attached to one or more hydroxyl groups. The structures of these compounds range from simple phenolic molecules to complex polymers [2]. Tea leaves contain about 10–30% polyphenols (dry leaf weight). Tea catechins are the major phenolic compounds in tea leaves and are associated with physiological properties, including anti-radical, antibacterial, and anti-aging activities [3].

**Figure 1:**
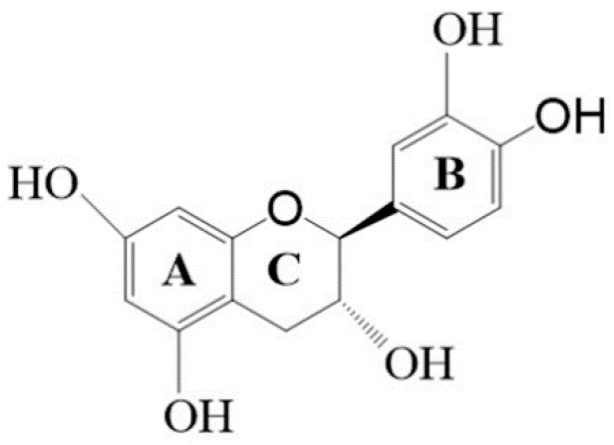
The molecular structure of (+)-catechin.

Irradiation, temperature, and pH are the dominant factors for the non-enzymatic browning of catechin-containing products [4]. Hence, the chemical transformation of catechins under different environments would be an important issue in the tea industry. Catechins are unstable in a solution and are easily oxidized [5]. Catechin is a flavan-3-ol with four phenolic hydroxyl groups, as shown in Figure 1. It is stable in an acidic pH, but unstable in neutral or alkaline situations under high-temperature treatments [6]. The esterification condensation of catechin and bicarboxylic acid in alkaline solutions with increasing temperatures and a yellowing of the reaction solution have been reported [7]. Catechin in an alkaline solution causes the cleavage of the heterocyclic ring (ring C) of flavan-3-ols via the oxidation of the ether bond when heated [6].

Both the quality and dosage of the light used for the photoreaction of catechin can affect the structural changes of catechin. Wilhelm-Mouton et al. reported that catechin and epicatechin, when irradiated by ultraviolet C (UVC) for 20 h in methanol, yielded products with the heterocyclic ring (ring C) opening of flavan-3-ols [8]. Liang et al. reported that the transparent solution of catechin became yellowish under blue light irradiation in neutral or alkaline conditions. The chromogenic catechin dimer was produced by the catechin photoreaction in an alkaline solution, and identified as a proanthocyanidin [9]. Hayashi et al. reported that the proanthocyanidins in fresh red rice were sensitive to light. Following photo-irradiation, the red rice color deepened, suggesting that the color change phenomenon was achieved via the formation of intramolecular bonds within proanthocyanidin, by oxidation [10].

Catechins are considered as effective free-radical scavengers and are one of the main healthy components of tea beverages. It has been reported that the catechins in canned or PET-bottled tea drinks are rather unstable during the manufacturing process [11]. Green tea infusions turning brown is an important issue in the tea industry and was shown to be as a result of the oxidation of tea polyphenols [4]. In bottled tea beverages, ascorbic acid is usually added for antioxidation, while pH adjustment is achieved by adding sodium bicarbonate. Ascorbic acid is an anti-oxidative reagent and a dienol compound. It is uncertain, however, whether a peroxidation reaction or the production of the free radical species occurs during the browning of green tea. It would be interesting to study how the anti-oxidative reagents alleviate the unstable situation of catechin in the browning processes.

The Folin–Ciocalteu method is commonly used as an in vitro assay for total polyphenol analysis [12]. Polyphenols are unstable in an alkaline solution. A Folin–Ciocalteu reagent reacting with polyphenols and the reduction of Mo^6+^ to Mo^5+^ through an electron-transfer mechanism under alkaline conditions has been measured [13]. Catechins undergo oxidation by losing hydrogen atoms with quinone-oxidized products, and semiquinone radical intermediates are generated in the reaction solution [14,15]. Catechin is easily oxidized and subjected to the electron-transfer mechanism in an alkaline solution. It would be of interest to examine the electron transfer and the O_2_•^−^ yielded via catechin photoreaction under blue light irradiation in aerobic photo-oxidative processes.

Reactive oxygen species (ROS), including hydrogen peroxide (H_2_O_2_), the hydroxyl radical (•OH), the superoxide anion radical (O_2_•^−^), and the peroxyl radical (ROO•), are reactive in general [16]. O_2_•^−^ is an intermediate generated during oxidation or reduction, and it causes tissue damage, inflammation, and atherosclerosis, in addition to aging the cells [17,18]. Many methods, both direct and indirect, have been used for detecting activity in scavenging O_2_•^−^. However, direct assays are rare because they need special equipment, such as an electron paramagnetic resonance (EPR) spectrometer. Indirect methods for determining the activity in scavenging O_2_•^−^ are more widely used for biochemical analysis [19]. The generation of O_2_•^−^ from intermediates can be detected via nitro blue tetrazolium (NBT) reduction [20]. NBT is used as an indicating scavenger that is reduced by O_2_•^−^ and can be employed to determine the quantity of O_2_•^−^ [18,21]. 

Many natural molecules are sensitive to visible light (400–800 nm) irradiation. Riboflavin is sensitive to blue light irradiation, as O_2_•^−^ is generated from the photolysis of riboflavin by electrons transferred from the ribityl side chain [22]. Liang et al. reported that the photochemical treatment of riboflavin or riboflavin-5′-phosphate (FMN) with blue light via electron transfer with a high quantum yield generated O_2_•^−^ that degraded crystal violet [23] and led to the inactivation of *Escherichia coli* (*E. coli*) and methicillin-resistant *Staphylococcus aureus* (MRSA) through ROS formation [24,25,26]. Catechin is sensitive to blue light in neutral or alkaline solutions [9]. It would be of interest to examine the inactivation of microorganisms by ROS formation via the catechin photoreaction under blue light irradiation in the aerobic photo-oxidative processes.

*A. baumannii,* a Gram-negative pathogenic colonizer, is found everywhere in soil, water, and on the skin of humans. It has been reported that *A. baumannii* is increasingly becoming a common nosocomial pathogen in intensive-care units (ICUs) worldwide [27]. The wide spread of *A. baumannii* and bacteria that can survive for up to one month on dry surfaces explains the difficulty of controlling this nosocomial infection [28]. *A. baumannii* is a human pathogen that produces a wide range of toxins accompanied by various symptoms. It is also one of the nosocomial pathogens causing wound infections related to serious diseases such as bacteraemia, meningitis, pneumonia, and urinary tract infections [29]. 

The adaptability of *A. baumannii* contributes to its widespread presence in the environment. This organism is generally intrinsically resistant to a number of commonly used antibiotics, such as aminopenicillins, first- and second-generation cephalosporins, and chloramphenicol [30]. On the other hand, antimicrobial drugs, carbapenems, have played an important role in the therapy for *A. baumannii* invasion [31]. However, it has also been reported that many infections are caused by CRAB or even extensively drug-resistant (XDR) strains, for which effective therapy is not well established [31]. As the development of new antibiotics for clinical use usually takes decades, alternative therapies that can meet hygienic requirements and sterilize the environmental and human sources of *A. baumannii* and CRAB are important.

Bacteria are intrinsically resistant towards antibiotics with a high efficiency during evolution, and there are different approaches that have been proposed to solve this dilemma. One novel approach is to use the antibacterial photodynamic inactivation of bacteria (aPDI) [32]. The application of aPDI to *A. baumannii* requires the use of a visible or UV light source for the inactivation of *A. baumannii*, such as methylene blue [33], PEI-c_e6_-conjugate [34], porphyrin [35], TiO_2_ [36], toluidine blue O [37], and ZnO nanoparticles [38]. Methylene blue and toluidine blue O are phenothiazinium dyes, and methylene blue is a hazardous substance. The effects of cationic hydrophilic porphyrin photoreaction by violet light (407 nm) irradiation on the viability of *A. baumannii* were examined [35]. Nanoparticle and UV irradiation are considered hazardous. The wavelength of UV or violet light is shorter than that of blue light. Lights of shorter wavelengths and, therefore, of higher energy cause a higher degree of damage to cells. Catechin is a flavan-3-ol, a type of natural phenolic compound and an antioxidant. It would be of interest to examine a promising, safe, and simple photosensitizer via catechin photoreaction under blue light (465 nm) irradiation.

Using chromatography and photospectrometry techniques, this study examines the changes in catechin hydrate under blue light irradiation with ascorbic acid added in a photoreaction system. Specifically, the detection of O_2_•^−^ generation and *A. baumannii* and CRAB inactivation during the catechin hydrate photoreaction were investigated.

## 2. Results

### 2.1. Effects of Blue Light Irradiation on Catechin Hydrate Treated with Ascorbic Acid 

**Figure 2:**
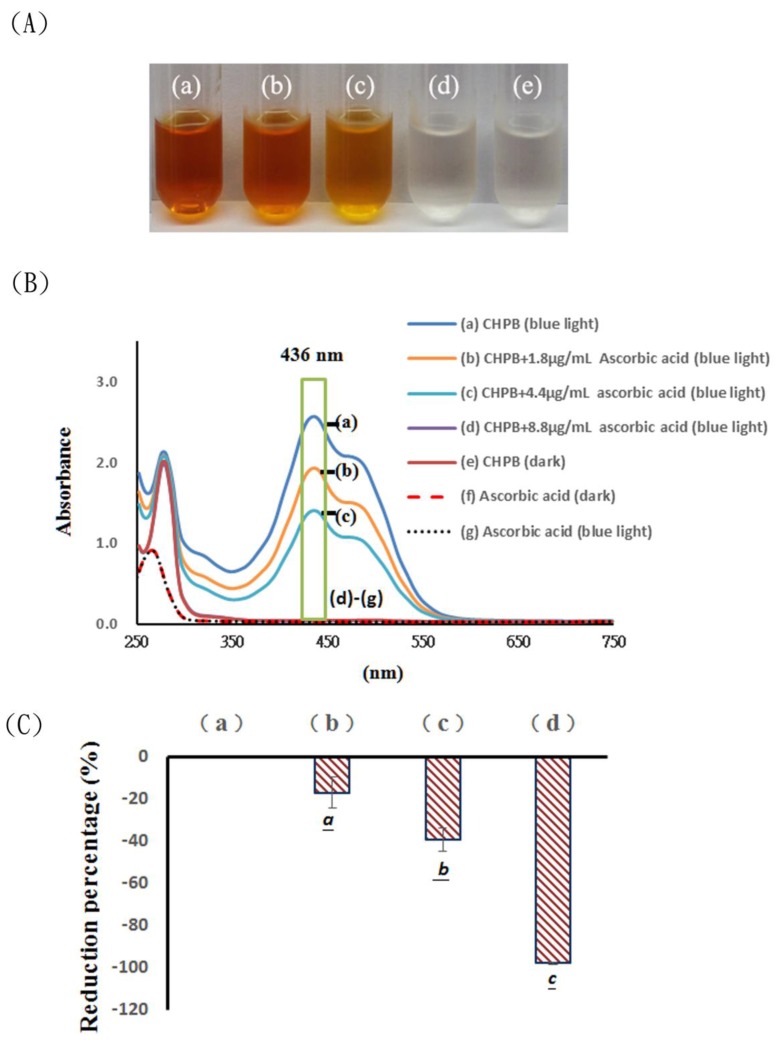
(**A**) Color changes of 290 μg/mL (catechin hydrate in 0.1 M phosphate buffer solution) CHPB treated (**a**) without and with (**b**) 1.8, (**c**) 4.4, and (**d**) 8.8 μg/mL of ascorbic acid, respectively, under blue light irradiation at 2.0 mW/cm^2^ for 120 min, and (**e**) in the dark. (**B**) The absorption spectra of 290 μg/mL CHPB treated (**a**) without and with (**b**) 1.8, (**c**) 4.4, and (**d**) 8.8 μg/mL of ascorbic acid, respectively, under blue light irradiation at 2.0 mW/cm^2^ for 120 min, and (**e**) in the dark. The absorption spectra of 17.6 μg/mL ascorbic acid solutions treated (**f**) without and (**g**) with blue light irradiation at 2.0 mW/cm^2^ for 120 min. The reaction solutions were measured in the spectral range of 250–750 nm. (**C**) The effects of ascorbic acid on absorbance at 436 nm. The reduction percentage at 436 nm of 290 μg/mL CHPB treated (**a**) without and with (**b**) 1.8, (**c**) 4.4, and (**d**) 8.8 μg/mL of ascorbic acid, respectively, under blue light irradiation at 2.0 mW/cm^2^ for 120 min. Data are represented by mean ± standard deviation (SD), where *n* = 3. Statistical differences (*p* < 0.05) between groups are indicated by different letters below each bar.

Catechin hydrate in a 0.1 M phosphate buffer solution (CHPB) under blue light (465 nm) irradiation was examined. The effects of blue light and ascorbic acid on the color and spectra changes of CHPB were studied. CHPB in an alkaline condition became yellowish under blue light irradiation, as shown in Figure 2A(a). CHPB at pH 8, treated with 1.8, 4.4, and 8.8 μg/mL of ascorbic acid under blue light irradiation at 2.0 mW/cm^2^ for 120 min, exhibited different levels of color. However, for 290 μg/mL CHPB treated with 8.8 μg/mL ascorbic acid under blue light irradiation at 2.0 mW/cm^2^ for 120 min, the solution appeared to be transparent, as shown in Figure 2A(d). As shown in Figure 2A(e), without light treatment, 290 μg/mL CHPB at pH 8 appeared transparent.

The spectra of the 290 μg/mL CHPB treated with or without ascorbic acid under blue light irradiation at pH 8, is shown in Figure 2B. It is observed that the CHPB under blue light treatment at pH 8 has two bands at 280 and 436 nm, as shown in Figure 2B(a). The CHPB treated with 1.8, 4.4, and 8.8 μg/mL of ascorbic acid under blue light irradiation at 2.0 mW/cm^2^ for 120 min exhibited different levels of absorption spectra. However, when CHPB was treated with 8.8 μg/mL ascorbic acid under blue light irradiation at 2.0 mW/cm^2^ for 120 min, only one peak at 280 nm, attributed to catechin, was found, in Figure 2B(d). In CHPB at pH 8, one peak at 280 nm was found in the spectrum of the dark control, in Figure 2B(e). The spectral changes of CHPB in the dark and CHPB treated with 8.8 μg/mL ascorbic acid under blue light irradiation were not significant. 

The spectra of ascorbic acid treated with blue light irradiation at pH 8 are shown in Figure 2B(f). In 17.6 μg/mL ascorbic acid solution at pH 8, one peak at 264 nm was found in the spectrum of the dark control. The spectral changes in ascorbic acid treated with blue light irradiation were not significant, as shown in Figure 2B(g).

The absorbance of the catechin solution at 436 nm was significantly increased by blue light irradiation. By quantitating the absorbance at 436 nm, the extent of the decrease at this wavelength upon the addition of ascorbic acid was noticed, with the reduction percentages being 17.1, 39.2, and 97.9 for 1.8, 4.4, and 8.8 μg/mL of ascorbic acid added under blue light irradiation, respectively (Figure 2C). The results show that changes in the catechin can be produced by photo-oxidation under blue light irradiation, and that ascorbic acid is an anti-oxidative reagent that can inhibit the formation of chromogenic catechin.

### 2.2. HPLC Analysis of CHPB Treated with Blue Light Irradiation and Ascorbic Acid 

**Figure 3:**
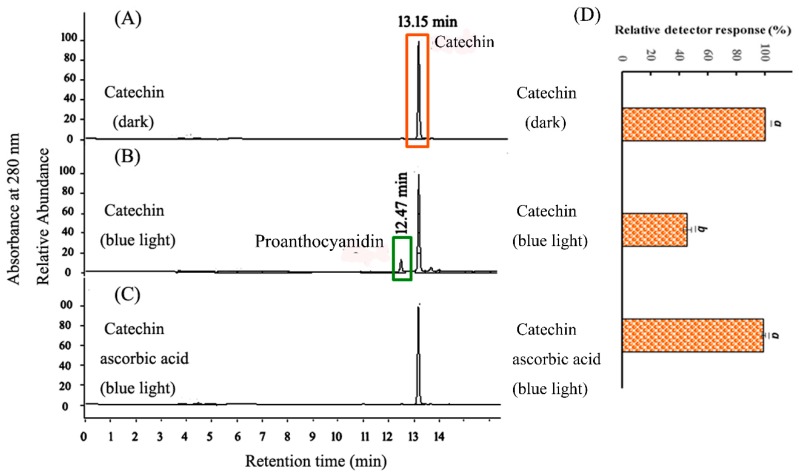
Chromatograms of HPLC-photodiode-array detector (DAD) analysis of (**A**) 290 μg/mL CHPB (pH 8) in the dark; (**B**) 290 μg/mL CHPB treated without and (**C**) with 8.8 μg/mL ascorbic acid, respectively, followed by blue light irradiation at 2.0 mW/cm^2^ for 120 min. (**D**) The relative contents of catechin under A, B, and C treatments. Data are represented by mean ± SD, where *n* = 3. Statistical differences (*p* < 0.05) between the groups are indicated by different letters above each bar.

As shown in Figure 3, the chromatograms of CHPB treated with blue light irradiation and ascorbic acid were measured by a photodiode-array detector (DAD) at 280 nm. For the CHPB at pH 8, a single chromatographic signal was found at 13.15 min, as shown in Figure 3A. After blue light irradiation, two peaks, namely catechin (at 13.15 min) and the photoreaction product of catechin (at 12.47 min), were observed, in Figure 3B. The catechin solution was identified at 13.15 min and confirmed by its mass spectra, with the major ion fragment being *m*/*z* 289. The photoreaction product of catechin was identified at 12.47 min, with the major ion fragment being *m*/*z* 577 (data not shown). The signals of the mass spectra were identified as the quasi-molecular cations [M − H]^−^. The molecular weights of catechin and the photoreaction product of catechin are 290 and 578 Da, respectively. A previous study suggested the photoreaction product of catechin to be a catechin dimer, which is a dimeric B-type proanthocyanidin [9]. The chromatogram of blue-light-treated 290 μg/mL CHPB and 8.8 μg/mL ascorbic acid solutions at pH 8 showed a single chromatographic signal at 13.15 min, as seen in Figure 3C. The relative catechin contents identified by the detector response evaluation were detected at 100%, 46%, and 99% for CHPB kept in the dark, treated with blue light, and treated with 8.8 μg/mL ascorbic acid under blue light irradiation at 2.0 mW/cm^2^ for 120 min, respectively (Figure 3D). The results show that ascorbic acid inhibited the formation of the catechin dimer.

### 2.3. Probing of Radicals in the Photoreaction of CHPB

The generation of O_2_•^−^ from the intermediates during the riboflavin photolysis in an aqueous solution was detected using NBT reduction, as previously described [20]. As shown in Figure 2 and Figure 3, the formation of the catechin dimer was inhibited by the anti-oxidant reagent, and the processing of catechin under blue light irradiation was able to act as photosensitive oxidation. In this study, O_2_•^−^ was analyzed using the catechin/NBT system. The O_2_•^−^ generated reduced NBT to form formazan, which can be detected at 560 nm. The CHPB treated with blue light photoreaction was able to generate O_2_•^−^. 

**Figure 4:**
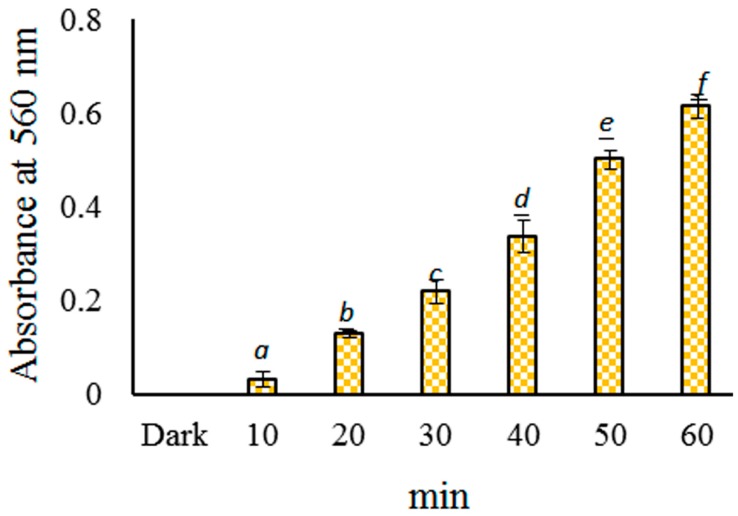
Effects of 290 μg/mL CHPB on nitro blue tetrazolium (NBT) reduction by blue light-emitting diode (LED) irradiation at 2.0 mW/cm^2^ for 10–60 min. Data are represented by mean ± standard deviation, where *n* = 3. Significant differences (*p* < 0.05) between each treatment are indicated by the different letters above the bar.

The reductions in catechin/NBT upon irradiation by a blue LED at 2.0 mW/cm^2^ for 10, 20, 30, 40, 50, and 60 min are shown in Figure 4. As shown in Figure 4, the photochemical effect of NBT reduction in CHPB was increased, along with the reaction time under blue light irradiation at a pH of 7.8. O_2_•^−^ could be produced from CHPB under blue light irradiation in an alkaline solution in this study.

### 2.4. Viability of A. baumannii under CHPB Photoreaction 

The inactivation of microbes induced by ROS from riboflavin and FMN photolysis have been reported [24,25,26]. The effects of CHPB photoreaction by blue light irradiation on the viability of *A. baumannii* were examined in this study. 

**Figure 5:**
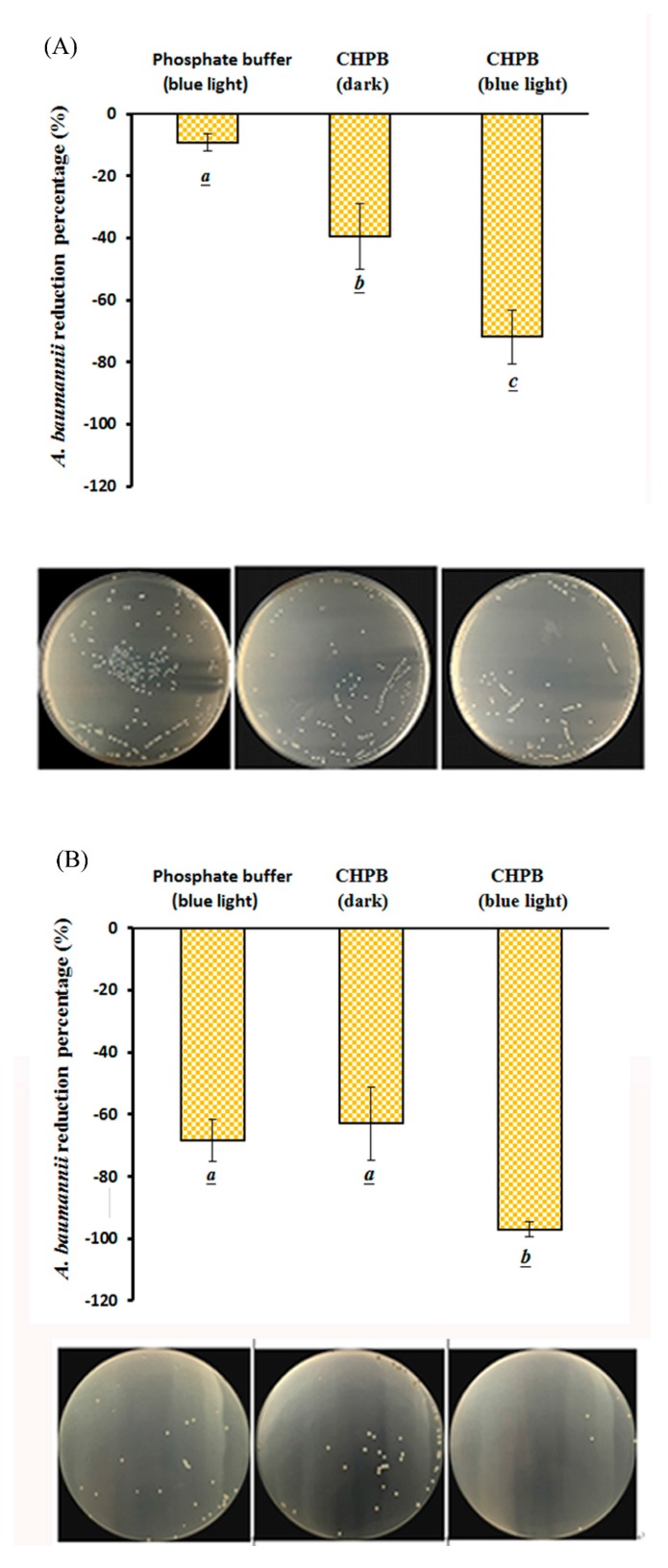
Effects of photochemical treatments on the viability of *A. baumannii* treated with (**A**) 145 μg/mL CHPB (pH 7.8) under blue light irradiation at 1.0 mW/cm^2^ for 60 min and (**B**) 290 μg/mL CHPB (pH 7.8) under blue light irradiation at 2.0 mW/cm^2^ for 120 min. Data are represented by mean ± SD, where *n* = 4. Statistical differences (*p* < 0.05) between groups are indicated by the different letters below each bar.

As shown in Figure 5A, the reduction percentage of *A. baumannii* increased with the addition of CHPB. The reduction percentage of *A. baumannii* inactivated with CHPB under blue light irradiation was significant (*p* < 0.05). According to Figure 5A, a 9.2% inactivation rate of *A. baumanni* was achieved without CHPB under blue light irradiation at 1.0 mW/cm^2^ for 60 min (with an energy dose of 3.8 J/cm^2^), and a 39.5% inactivation rate was achieved with a 145 μg/mL CHPB treatment for 60 min. However, a 71.8% inactivation rate of *A. baumanni* was achieved with 145 μg/mL CHPB under blue light irradiation at 1.0 mW/cm^2^ for 60 min, as shown in Figure 5A. 

As shown in Figure 4, the absorbance of the NBT reduction increased with the generation of O_2_**•^−^** by the photochemical system in the presence of CHPB. In this study, the detection of O_2_•^−^ and the inactivation of *A. baumanni* were examined during the photoreaction of catechin. Catechin is unstable in an alkaline solution and exhibits photosensitive oxidation during blue light irradiation, with O_2_•^−^ being produced from catechin as an intermediate. Under critical circumstances, the O_2_**•^−^** produced from the catechin photoreaction could enhance the inactivation rate of *A. baumanni*.

According to Figure 5B, a 68.4% inactivation rate of *A. baumanni* was achieved without CHPB under 2.0 mW/cm^2^ of blue light irradiation for 120 min (with an energy dose of 14.4 J/cm^2^). There is no significant difference (*p* = 0.87) in the inactivation rate of *A. baumanni* by blue light irradiation in the absence of CHPB at 2.0 mW/cm^2^ for 120 min, or the presence of 290 μg/mL CHPB in the dark, as shown in Figure 5B. The inactivation rate of *A. baumanni* was increased to 97.1% by the same irradiation conditions in the presence of 290 μg/mL CHPB, while the viable colony levels of the surviving *A. baumanni* were below 15 colony forming unit (CFU)/plate, as shown in Figure 5B. After a series of dilutions, 290 μg/mL the CHPB-treated photoreaction inhibited the growth of *A. baumanni* by 2 to 3 log, under blue light irradiation at 2.0 mW/cm^2^ for 120 min in this study. 

### 2.5. Viability of CRAB under the CHPB Photoreaction

**Figure 6:**
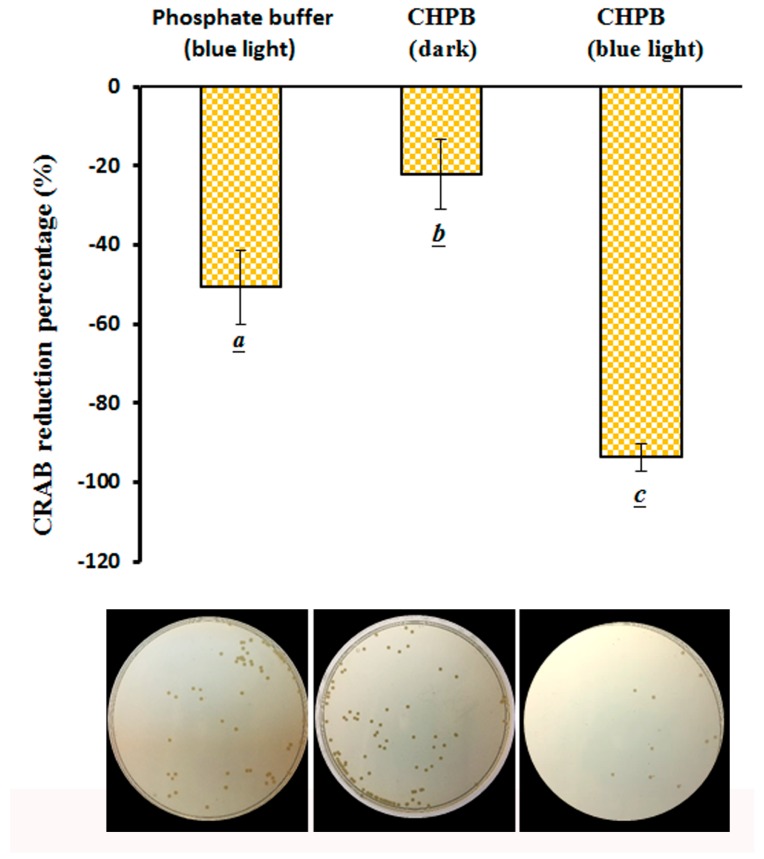
Effects of photochemical treatments on the viability of carbapenem resistant *A. baumannii* (CRAB) treated with 290 μg/mL CHPB (pH 7.8) under blue light irradiation at 2.0 mW/cm^2^ for 120 min. Data are represented by mean ± SD, where *n* = 7. Statistical differences (*p* < 0.05) between groups are indicated by the different letters below each bar.

The effects of the CHPB photoreaction (by blue light irradiation at 2.0 mW/cm^2^ for 120 min) on the viability of CRAB were examined. As shown in Figure 6, a 50.7% inactivation rate of CRAB was achieved, without CHPB, under blue light irradiation at 2.0 mW/cm^2^ for 120 min and a 22.2% inactivation rate was achieved with a 290 μg/mL CHPB treatment for 120 min. The photochemical effect of blue light irradiation at 2.0 mW/cm^2^ for 120 min in the presence of 290 μg/mL CHPB achieved a 93.8% inactivation rate for CRAB, while the viable colony levels of the surviving CRAB were 0–20 CFU/plate, as shown in Figure 6. After a series of dilutions, the CHPB-treated blue light irradiation processes inhibited the growth of CRAB by 2 to 3 log, with an energy of 14.4 J/cm^2^ (equivalent to 2.0 mW/cm^2^) for 120 min in the presence of 290 μg/mL CHPB in this study.

## 3. Discussion

Catechins account for the largest amount of polyphenols in green tea. Tea infusions turning brown is an important factor influencing the shelf life of canned or PET-bottled tea drinks. In Taiwan, 2.4 million bottles of green tea beverages were abandoned, according to the Food and Drug Administration (2010), because of storage factors, which may result in the natural degradation of catechins [6,9]. Under blue light irradiation, the transparent solution of catechin became yellowish, as shown in Figure 2, while irradiation with green and red lights led to insignificant changes [9]. There was a 53% decrease in catechin and a chromogenic catechin dimer was generated following treatment under blue light irradiation for 120 min, in Figure 3. As in Figure 3, 290 μg/mL CHPB in the dark and CHPB treated with 8.8 μg/mL ascorbic acid under blue light irradiation showed almost the same catechin content, and the chromogenic catechin dimer was not observed. As shown in Figure 2 and Figure 3, the addition of ascorbic acid is required to inhibit photosensitized oxidation for ROS formation and suppress the oxidation of catechin. Such processes preserve the natural flavor and stability when manufacturing tea beverages.

The conformational changes of catechin may affect the chemical properties in the solution. Following the photoreaction treatment, the scavenging O_2_•^−^ activity and total phenolic contents of catechin treated with or without blue light irradiation showed no significant changes in neutral or alkaline solutions [9]. As shown in Figure 4, the generation of O_2_•^−^ from the intermediates during the catechin photoreaction in aqueous solutions at pH 7.8 was detected using the NBT reduction method. Phenolic compounds are unstable in an alkaline solution. When using the Folin–Ciocalteu method for a total polyphenol analysis, phenolic compounds react with the Folin–Ciocalteu reagent and change color through an electron transfer mechanism under an alkaline environment [13]. Catechins are unstable under UV irradiation, which ranges from 100 to 400 nm in wavelength [9]. In CHPB at pH 8, one peak at 280 nm was found in the spectrum of the dark control, as shown in Figure 2B(e). Catechin is a flavan-3-ol that possesses two benzene rings and shows a strong absorption capacity in the π electrons of benzene rings under UV light irradiation. Shi et al. reported that, after being irradiated by ultraviolet B (UVB) for 360 min, the catechin in water or ethanol solutions exhibited a low photostability with the generation of several new photoproducts, which is due primarily to photo-induced electron transfers [3]. Lights with shorter wavelengths, such as UVB, that have a high energy and can be strongly absorbed by the catechin, may cause photochemical reactions. The CHPB under blue light treatment at pH 8 became yellowish, and two bands at 280 and 436 nm occurred, as shown in Figure 2B, with the chromogenic catechin being proanthocyanidin [9]. Liang et al. reported that the reduction percentages of catechin with blue light illumination were 17.3, 43.8, and 63.9%, calculated at pH 6, 7, and 8, respectively [9]. In the Folin–Ciocalteu method for total polyphenol analysis, phenolic compounds easily dissociate a phenolic proton, leading to a phenolate anion that acts as a reductant via an electron transfer mechanism in alkaline aqueous circumstances [7]. The pH of 290 μg/mL catechin hydrate in pure water is 6.0, and catechin is stable in acidic solutions. However, in this study, catechin was not stable under neutral or alkaline aqueous conditions with the illumination of blue light. As shown in Figure 4, this study reports aerobic photo-oxidative processes for catechin under blue light irradiation in an alkaline solution, which causes the generation of O_2_•^−^ by photosensitized oxidation via an electron transfer mechanism.

**Figure 7:**
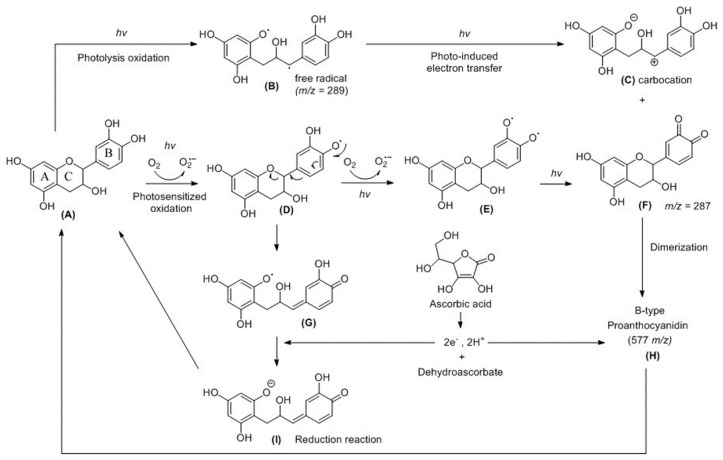
Proposed scheme for the photoreaction mechanism of catechin and interactions of intermediates with ascorbic acid.

Based on the results of the present study, we propose a catechin photoreaction mechanism under the influence of ascorbic acid, as shown in Figure 7. Two pathways are proposed for the photo-oxidation of flavan-3-ols, as ollows: singlet molecular oxygen directly reacts with the substrate, in addition to the production of radicals upon the reaction with oxygen [39]. It has also been suggested that the autoxidation of catechins by losing hydrogen atoms occurred with quinone-oxidized products, with the semiquinone radical intermediates and O_2_•^−^ being generated in the reaction solution [14,15]. The oxidation of catechin under blue light irradiation is the one-electron oxidation of the B ring of catechin by molecular oxygen to generate O_2_•^−^. The –OH bond of the heterocyclic ring was excited via photosensitized oxidation, leading to the formation of a quinone compound at *m*/*z* = 287, as shown in Figure 7F. The heterocyclic ring (ring C) of catechin was preferentially opened via the photolytic cleavage of the ether bond with low bond dissociation energies, resulting in the generation of free radicals at *m*/*z* = 289, as shown in Figure 7B [3]. The neutral radicals can be ionized in a polar solvent via a photo-induced electron transfer reaction, with the product being a transient carbocation of catechin, as shown in Figure 7C [40]. In Figure 7F, B-type proanthocyanidin at *m*/*z* = 577 can be generated from the condensation of the quinone compound and a carbocation intermediate of catechin [41,42,43]. Ascorbic acid is used as an antioxidant because of its strong reducing power. In Figure 7H, the disconnection within proanthocyanidin is achieved by the cleavage of C–C bonds between the two catechin moieties [42] via the electrons and hydrogen ions from ascorbic acid. At the same time, the semiquinone radical is quenched by the strong antioxidant, as shown in Figure 7G, suggesting that the reduction reaction can be enhanced in the presence of ascorbic acid.

Oxidative stress can cause damage to all types of biomolecules, including nucleic acids, proteins, and lipids (lipid peroxidation) [44]. A 45.1% inactivation rate of *E. coli* was achieved with 290 μg/mL CHPB under blue light irradiation at 2.0 mW/cm^2^ for 120 min (data not shown). FMN is sensitive to blue light. Under the same irradiation conditions, a 60.1% inactivation rate of *E. coli* was achieved with 4.6 μg/mL FMN in our previous study [25]. The supercoiled plasmid DNA was expanded to react with the ROS generated to inspect the extent of DNA cleavage [24,25]. Excessive DNA strand breakage might deplete cellular ATP and NAD^+^ levels, interfere with ATP synthesis, and even lead to cell death [26,44]. After being lightly photo-energized, FMN (riboflavin-5′-phosphate) is converted into an oxidized form, and O_2_•^−^ or singlet oxygen is generated [25,26]. The catechin photochemical effect of blue light irradiation was less than the FMN photochemical effect, in terms of the inactivation of *E. coli*. 

As shown in Figure 5, a 62.9% inactivation rate of *A. baumanni* was achieved with 290 μg/mL CHPB treatment for 120 min. Previous research reported that catechin hydrate has antimicrobial activity against *S. aureus* clinical strains, with minimal inhibitory concentrations (MICs) ranging from 256 to 2048 µg/mL, but the mechanism of the catechin hydrate effect on the bacterial cells is still unknown [45]. It has been reported that catechin hydrate can inhibit urease in *Staphylococcus saprophyticus* strains [46]. It has also been shown that the antibacterial activity of catechin might reduce the biosynthesis of the virulence factors by affecting quorum-sensing mechanisms in *Pseudomonas aeruginosa* [47].

The photochemical effect of blue light irradiation at 1.0 mW/cm^2^ for 60 min (with an energy dose of 3.6 J/cm^2^) without CHPB added achieved nearly a 9.2% inactivation rate for *A. baumanni,* and the rate for blue light irradiation at 2.0 mW/cm^2^ for 120 min reached approximately 68.4% (at an energy dose of 14.4 J/cm^2^) in Figure 5. It has been reported that the utility of antimicrobial therapy for multidrug-resistant *A. baumanni* infection in a mouse-burned model suggests that blue light significantly reduced the bacterial burden in mouse burns by endogenous porphyrins within *A. baumannii* cells [48,49]. Endogenous intracellular porphyrins are similar to photosensitizers, in that they can be excited by blue light and, in turn, create ROS, causing cell death [26]. 

Catechins are a class of polyphenolic flavonoids composed mainly of catechin, epi-catechin, epicatechin gallate (ECG), epigallocate catechin (EGC), and epigallocatechin gallate (EGCG) [50]. It has been reported that EGCG has antimicrobial effects against multi-resistant clinical isolates of *A. baumannii*. The isolates of *A. baumannii* were inactivated by EGCG incubation for 300 min, and the MIC_50_ and MIC_90_ were 312 and 625 μg/mL, respectively [27]. As shown in Figure 5 and Figure 6, the catechin effect was less than the EGCG effect in terms of the inactivation rate of *A. baumannii* and CRAB. While the 290 μg/mL CHPB was treated with blue light irradiation, 97.1% and 93.8% inactivation rates of *A. baumanni* and CRAB were achieved, respectively. Blue-light-induced O_2_•^−^ formation from catechin in neutral or alkaline aqueous solutions was able to enhance the inactivation rate of *A. baumannii* and CRAB in this study. The bactericidal agent should inhibit bacterial growth at a rate of at least 3 log. For the bactericidal activity condition, the CHPB concentrations and light doses were both raised to increase the *A. baumannii* inactivation rate in the study. The effects of 580 μg/mL CHPB photoreaction by blue light irradiation at 2.0 mW/cm^2^ for 240 min on the viability of *A. baumannii* were examined. After a series of dilutions, 580 μg/mL CHPB-treated photoreaction inhibited the growth of *A. baumannii* by 4–5 log, under blue light irradiation at 2.0 mW/cm^2^ for 240 min (data not shown). Additionally, increasing the temperature of the photoreaction system may cause a higher degree of CHPB photoreaction and more O_2_•^−^ formation. The photoreaction system temperature was kept at 25 ± 2 °C, and in the presence of 290 μg/mL CHPB, the effects of blue light irradiation at 2.0 mW/cm^2^ for 120 min on *A. baumannii* viability were shown to inhibit the growth of *A. baumannii* by 5–6 log (data not shown). Blue light can, therefore, be considered a novel light source for CHPB photosensitized reactions, as CHPB acts as a promising photosensitizer.

## 4. Materials and Methods

### 4.1. Chemicals

Acetonitrile, ascorbic acid, (+)-catechin hydrate, methanol, mono-potassium phosphate, and potassium dihydrogen phosphate were purchased from Sigma-Aldrich (St. Louis, MO, USA). NBT was obtained from Bio Basic, Inc. (Markham, ON, Canada). Ultra-pure water by the Milli-Q system was used as a solvent throughout this study.

### 4.2. Setup of Irradiation Units

**Figure 8:**
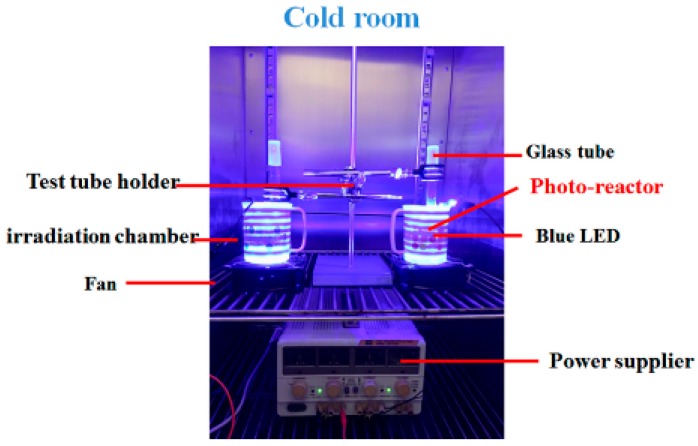
Set-up of the photoreaction system.

As used previously [24,25,26], the photoreaction system consisted of an irradiation chamber (opaque plastic with a height and diameter of 8 and 7 cm, respectively) and a power supply, as shown in Figure 8. The reaction solution in a glass tube was placed at the top end of the chamber. Six DC 12 V 5050 blue LED lamps (vitaLED Technologies Co., Tainan, Taiwan) were placed inside the irradiation chamber. The power supply (YP30-3-2, Chinatech Co., New Taipei City, Taiwan) and a solar power meter (TM-207, Tenmars Electronics Co., Taipei, Taiwan) were utilized to control light irradiance. The blue LED lamps were placed inside the chamber. As the temperature can be increased during the irradiation experiments, the photoreaction system was placed in a cold room with the temperature kept at 10 ± 1 °C by an infrared thermometer (MT 4, Raytek Co., Santa Cruz, CA, USA). The wavelength of the emitted maxima of the blue light was 465 nm, according to a UV-VIS miniature fiber optic spectrometer (USB4000 UV/Vis, Ocean Optics, Dunedin, FL, USA), and the W_1/2_ (spectral width at half height) was 26 nm. 

### 4.3. Effects of Ascorbic Acid on the CHPB Treated with Blue Light Irradiation

The effects of ascorbic acid on the changes in catechin treated with blue light irradiation were determined by an ELISA reader. In short, (A), or 290 μg/mL CHPB (pH 8) kept in the dark, was used as a standard. In (B), 290 μg/mL CHPB (pH 8) was irradiated by blue light at 2.0 mW/cm^2^ for 120 min. In (C), 290 μg/mL CHPB (pH 8) was treated with 1.8, 4.4, and 8.8 μg/mL of ascorbic acid, respectively, under blue light irradiation at 2.0 mW/cm^2^ for 120 min. The absorbance of the catechin solution was monitored at a range of 250–750 nm by an ELISA reader (i.e., Thermo Fisher Scientific Multiskan spectrophotometer).

### 4.4. HPLC-DAD Analysis of CHPB Treated with Blue Light Irradiation

Catechin and its relevant photoproducts were examined using HPLC analysis, as described previously [6,9]. In short, (A), or 290 μg/mL CHPB (pH 8) kept in the dark, was used as a standard. In (B), 290 μg/mL CHPB (pH 8) was irradiated by blue light at 2.0 mW/cm^2^ for 120 min. In (C), 290 μg/mL CHPB (pH 8) was treated with 8.8 μg/mL ascorbic acid under blue light irradiation at 2.0 mW/cm^2^ for 120 min. The analysis of catechin and its relevant photoproducts were carried out using an Agilent 1200 series HPLC system (Agilent Technologies, Palo Alto, CA, USA) equipped with a G1315A DAD. The reaction solutions were separated using an Agilent Poroshell 120 EC-C_18_ column (2.7 µm, 4.6 mm id × 150 mm, Agilent Technologies, Palo Alto, CA, USA). The CHPB was filtered through a 0.45 μm filter (Millipore) before use. The DAD detector was set at 280 nm to acquire chromatograms. The separation was achieved with a mobile phase consisting of 0.1% formic acid as solvent A and acetonitrile as solvent B, with gradient elution performed as follows. The linear gradient started at 0–3 min with 99–90% of solvent A; 3–10 min with 90–80% of solvent A; 10–16.5 min with 80–75% of solvent A; and 16.6–20 min with 50–0% of solvent A. The final mobile phase was programmed to 99% of solvent A, from 20 to 23 min. Samples of 10 μL were subsequently injected at a flow rate of 500 μL/min. The phosphate buffer was prepared from mono-potassium phosphate and potassium dihydrogen phosphate.

### 4.5. Detection of O_2_•^−^ Using an NBT Reduction Assay

NBT reduction was used as an indicator of O_2_•^−^ generation, and the assay was modified from the riboflavin photolysis assay, as previously described [20]. The effects of the CHPB photoreaction processes on O_2_•^−^ generation were inspected in this study. All of the chemical substances were freshly prepared before the experiments. First, 109.3 mg of L-methionine was added to 73.3 mL 0.1 M of potassium phosphate buffer solution (pH 7.8). After the L-methionine was dissolved, 10 mg NBT powder and 8 mL 2900 μg/mL CHPB (pH 7.8) were added to the solution. Then, the reactant was used. The concentrations of catechin hydrate, methionine, and NBT were 290, 1343, and 118 μg/mL in the reactant, respectively. The reactants were irradiated by blue light at 2.0 mW/cm^2^ for 10, 20, 30, 40, 50, and 60 min. The O_2_•^−^ generated from the photochemical reaction, which reduces NBT to form formazan, was detected at 560 nm.

### 4.6. Survival of A. baumannii *and CRAB* after the Photoreaction Processes of CHPB 

It has been reported that the photolysis of riboflavin or FMN under blue light treatment can be employed to inactivate *E. coli* or *S. aureus* by ROS upon photo-irradiation [24,25,26]. The effects of the CHPB photoreaction on *A. baumannii*, including a carbapenem resistant strain inactivation in the photoreaction system under blue light irradiation, were examined.

*A. baumannii* (BCRC Taxonomy ID: 10591T) and carbapenem resistant *A. baumannii* clinical isolate from the National Cheng Kung University Hospital (Taxonomy ID: BA005) were grown overnight in Tryptic soy broth (TSB) at 37 °C. The CRAB BA005 was a clinic wound isolate, not an epidemic strain. The CRAB BA005 was a single clone from this isolate. After overnight growth, 500 μL of *A. baumannii* or CRAB was loaded in a 1.5 mL centrifuge tube and diluted with 2-fold sterilized water. Cultures were grown to an optical density at 600 nm (OD_600_) of 0.5 (ca. 7.9 × 10^7^ CFU/mL) for *A.* *baumannii*. Following centrifugation at 10,000 rpm for 10 min, the supernatant was removed. One milliliter of potassium phosphate buffer (0.1 M) at pH 7.8 was added and mixed well. Then, the bacteria solution was diluted with CHPB (pH 7.8) and transferred to the glass tube for irradiation with blue light. In short, (A), or the bacteria in 0.1 M phosphate buffer solution (pH 7.8) kept in the dark, was used as a control. In (B), the bacteria in 145 or 290 μg/mL CHPB were kept in the dark. In (C), the bacteria in 0.1 M phosphate buffer solution (pH 7.8) were treated with blue light irradiation at 1.0 mW/cm^2^ for 60 min or 2.0 mW/cm^2^ for 120 min. In (D), the bacteria in 145 and 290 μg/mL CHPB were treated with blue light irradiation at 1.0 mW/cm^2^ for 60 min and 2.0 mW/cm^2^ for 120 min, respectively. For the dark control treatment, plates and tubes containing the bacterial phosphate solution were covered by thick aluminum foil. 

The photoreaction system was placed in a cold room (10 ± 1 °C). After irradiation, the 0.2 mL reaction solutions were diluted with sterilized water and transferred to LA plates for overnight growth at 37 °C. The survival of *A. baumannii* or CRAB following treatment was examined by counting the number of viable colony forming units (CFUs), following overnight incubation. The inactivation rate of *A. baumannii* or CRAB was calculated as the percentage of decrease (= [1 ‒ *T*/*C*] × 100%, where *T* is the number of CFUs of the test groups and *C* is that of the control in the dark). Thus, the reduction percentage was defined as the negative value of the inactivation rate.

### 4.7. Statistics

A one-way analysis of variance (ANOVA) was performed to identify the significant differences in more than three groups. When statistically significant differences were indicated, an unpaired Student’s t-test was used for further analysis. The results were expressed as mean ± standard deviation. A value of *p* < 0.05 indicates statistical significance.

## 5. Conclusions

The effects of blue light on the inactivation of pathogenic microorganisms with catechin hydrate photoreaction were examined. Blue light can be considered a novel light source for catechin hydrate photosensitized oxidation. Chromatographic analyses show that the chromogenic catechin dimer was generated from the photo-induced electron transfer of catechin through oxidation under blue light irradiation in an alkaline solution. The addition of ascorbic acid is required to inhibit the photosensitized oxidation for ROS formation and to suppress the oxidation of catechin. The photochemical treatment of catechin hydrate with blue light via photosensitized oxidation with quantum yield generated O_2_•^−^ was able to inactivate *A. baumannii* and CRAB by ROS formation. The catechin hydrate photoreaction with blue light irradiation was able to enhance the inactivation rate for *A. baumannii* and CRAB. Catechin photochemical treatment might be a simple and safe technique for hygienic decontamination.
